# Learning Subject-Generalized Topographical EEG Embeddings Using Deep Variational Autoencoders and Domain-Adversarial Regularization

**DOI:** 10.3390/s21051792

**Published:** 2021-03-04

**Authors:** Juan Lorenzo Hagad, Tsukasa Kimura, Ken-ichi Fukui, Masayuki Numao

**Affiliations:** 1 Graduate School of Information Science and Technology, Osaka University, Suita, Osaka 565-0871, Japan; 2 Institute of Scientific and Industrial Research, Osaka University, Ibaraki, Osaka 567-0047, Japan; kimura@ai.sanken.osaka-u.ac.jp (T.K.); fukui@ai.sanken.osaka-u.ac.jp (K.-i.F.); numao@sanken.osaka-u.ac.jp (M.N.)

**Keywords:** electroencephalography, emotion modeling, subject independence, domain generalization, variational autoencoder, domain adversarial network

## Abstract

Two of the biggest challenges in building models for detecting emotions from electroencephalography (EEG) devices are the relatively small amount of labeled samples and the strong variability of signal feature distributions between different subjects. In this study, we propose a context-generalized model that tackles the data constraints and subject variability simultaneously using a deep neural network architecture optimized for normally distributed subject-independent feature embeddings. Variational autoencoders (VAEs) at the input level allow the lower feature layers of the model to be trained on both labeled and unlabeled samples, maximizing the use of the limited data resources. Meanwhile, variational regularization encourages the model to learn Gaussian-distributed feature embeddings, resulting in robustness to small dataset imbalances. Subject-adversarial regularization applied to the bi-lateral features further enforces subject-independence on the final feature embedding used for emotion classification. The results from subject-independent performance experiments on the SEED and DEAP EEG-emotion datasets show that our model generalizes better across subjects than other state-of-the-art feature embeddings when paired with deep learning classifiers. Furthermore, qualitative analysis of the embedding space reveals that our proposed subject-invariant bi-lateral variational domain adversarial neural network (BiVDANN) architecture may improve the subject-independent performance by discovering normally distributed features.

## 1. Introduction

Emotions play an important role in daily life, not only in regulating human interactions but also in influencing our perception of the world around us. Computer systems may also benefit from learning and understanding human emotions, enabling them to facilitate smooth human–computer interactions, leading to an improved overall user experience [1]. We convey emotions in different ways: through facial expressions, vocal tone, or gestures. Modern sensors and data-processing techniques can convert these into data channels, which can be decoded by computer systems. Naturally, each data channel has its pros and cons. For example, features that appear to be the most natural for human observers might also be the modalities most prone to outside influence and the most likely to differ across cultures. This can hinder the identification of the true state of a subject, particularly when the subject knows it is being observed (i.e., observer effect). Thus, it may be beneficial to consider other signals, particularly those closer to the fundamental source of emotions, the brain. Electroencephalography (EEG) has gained considerable attention from researchers since it has advanced into a viable option for portable, affordable, and easy-to-use solutions for detecting emotions directly from the brain. However, the biggest challenge for existing EEG technologies is that the signal is very weak and is naturally subject to non-emotion related noise effects [2]. This noise can be internal, a natural effect of user variability, or external, caused by electrical signals from the rest of the body or the surrounding environment. The true extent of this noise is difficult to measure; however, studies [3] have shown that as much as half of EEG signals can be under the influence of facial expressions alone. Therefore, it is important to be able to extract the most stable brain emotion features from the EEG data to build a robust emotion model.

EEG is prone to certain types of noise; this necessitates the use of some combination of signal cleaning and enhancement techniques in the processing pipeline. The most common of these are signal filters designed to filter out nuisance signals from certain frequency bands not directly related to brain signals. Additionally, it is occasionally necessary to decompose and analyze the components of the cleaned signal to further improve the signal-to-noise ratio. Of these, some of the most commonly used are independent component analysis (ICA) [4], artifact subspace reconstruction (ASR) [5], common average reference, surface Laplacian, and principal component analysis (PCA) [6]. These techniques can be used to identify noise sources and remove them from the signal. However, most of these have the disadvantage of requiring manual intervention by the researcher to distinguish and remove the noise sources from the decomposed signal.

In recent years, similar forms of manual filtering and feature engineering have largely been made obsolete in other signal-processing fields, such as computer vision, due to the prominence of deep learning (DL). In response, recent studies in EEG emotion analysis have also explored machine learning as a strategy to automatically discover the most relevant features from the EEG signal through different DL techniques and architectures [7]. In particular, significant effort is being put toward developing models that can perform well across different subjects. This is one of the goals of the current work, to achieve improved generalizability by identifying subject-invariant features from EEG.

### 1.1. Machine Learning with EEG

Machine learning, particularly deep learning, offers to bypass some of the EEG feature extraction steps by discovering features directly from the data distributions. To leverage the full functional potential of DL, some studies propose building end-to-end pipelines for EEG. In one of the most prominent works [8], researchers proposed a specialized DL architecture with convolutional neural networks (CNNs) to learn directly from the raw EEG signals. To emulate the time-frequency filters normally used to extract band power features from EEG, they introduced a structured temporal and spatial convolution strategy that outperformed filter bank common spatial patterns (FBCSP) [9], the de facto standard method at the time. A more recent work proposed EEGNet [10], a similar architecture to the previous DeepConvNet, but with even more strongly specialized filters designed for EEG signal processing. Specifically, they introduced depthwise convolutional filters and pointwise filters to estimate bandpass filters, and they analyzed the features learned by their models on several EEG datasets. They found that the EEGNet model was successful in learning some filters that showed a good correlation with certain bandwidths.

While promising, a key limitation of these works is that learning the most basic generic filters, as is the case in image-based DL models, requires a large comprehensive training dataset. To alleviate this, end-to-end EEG models tend to feature fewer learnable parameters by limiting the number of layers and networks weights, and by constraining each layer to as few filters as possible to avoid overfitting. Even the DeepConvNet implementation in Schirrmeister et al. [8] is relatively shallow in comparison to most architectures used in image and audio analyses. A survey [11] found that larger DL models are not effective for EEG BCI applications since they are regularly outperformed by shallower implementations. This concurs with our findings in a previous work analyzing meditation [12], where a shallow model matched or even outperformed more complex deep models. This is likely the result of the relatively small size of available EEG datasets and the large number of parameters typically used in DL models. Modeling a complex phenomenon such as emotion may require a considerably large dataset or different methods of regularization to learn an ideal set of generic low-level features. In the interim, where neither a sufficiently large dataset for EEG nor a generic deep feature extractor is available, it may be prudent to opt for the standard frequency filtering methods to build robust high-level feature representations.

### 1.2. Topographic Models

Due to the complexity of learning end-to-end EEG models, many works have opted to build machine-learned emotion models from EEG topographs. This representation combines the time-frequency features of fast Fourier transform/power spectral density (FFT/PSD) filters with the spatial information of the EEG channel locations. This allows the modeling of the signal distribution across different regions of the brain. One of the earliest works to propose the use of spectral topography maps with DL was Bashivan et al. [13]. Their study used CNNs and recurrent neural networks (RNNs) to model the effects of mental workloads on EEG. Their experiments showed that deep models trained on EEG topography data could cope well with inter- and intra-subject differences. Our work is patterned on Bashivan et al. [13] in that we also use image-based representations of EEG; however, we forgo the use of long-term short-term memory (LSTM) models due to their high data requirements.

In a more recent work [14], the researchers proposed a similar bi-hemispheric model of EEG emotion; however, they used an end-to-end model based on domain adversarial training. This was a vast improvement from Schirrmeister et al. [8] in that features were learned automatically through DL. As input, they used differential entropy (DE) features passed to two feature extractors, one for each hemispheric EEG signal. Furthermore, three discriminators were used: one for each brain hemisphere and the other for the whole brain features. Thereafter, adversarial regularization was used to adapt the feature distribution of the trained source domains to each target user domain. As a result, their BiDANN model performed better than state-of-the-art approaches on the SEED dataset [15,16], another popular public EEG dataset, particularly on subject-independent tests. They have since refined their approach by implementing the input electrode streams as pairwise sub-networks organized into horizontal and vertical streams [17]. This allows their model to learn how EEG signals traverse over time and across different regions in the brain. This approach saw improvements in both subject-dependent and subject-independent tests. Both of these methods required domain-adversarial training or fine-tuning of the model to each target subject. They represent a step towards finding subject-invariant EEG features which could be the key to solving the subject generalization problem.

### 1.3. Subject-Invariant Feature Extraction

Regardless of the input feature, a key challenge in EEG-emotion studies is finding high-level features that are invariant across subjects. *Domain adaptation* and *domain generalization* approaches attempt to solve this problem by allowing models to optimize for features that can generalize across different domains [18]. Here a *domain* represents a probability distribution from which one can obtain samples used to train a model (e.g., training and testing). Domain adaptation aims to produce optimized models for the target domain by applying knowledge learned from a labeled source domain, while also leveraging unlabeled samples from the (possibly unlabeled) target domain. The problem of contextual variance is not unique to EEG studies. In fact, domain adaptation is currently a hot topic in the field of computer vision [18,19]. This is driven by realizations that along with the great learning potential and flexibility of deep learning models comes the increased propensity to overfit to a single sample distribution (i.e., the training set) [20].

On the other hand, domain generalization aims to learn a unified representation from a set of similar domains, with the intent of maximizing its usability on an unseen target domain. Unlike domain adaptation, domain generalization does not require adjusting the model to adapt to a new target, making it more appealing for systems designed for general use. In this work, we propose a robust domain-generalized EEG emotion architecture that combines latent space regularization based on variational auto-encoders (VAEs) with subject adversarial regularization.

## 2. Literature Review

While most domain adaptation techniques were originally intended for other signal processing applications such as image processing and sensor drift [21], a growing number of these techniques are gradually being applied successfully to EEG analysis. Roy et al. [7] provides a comprehensive survey of recent advancements in DL for EEG analysis, while Lan et al. [22] provides a good benchmark of domain adaptation methods applied to EEG-emotion data. In this section, we discuss a number of recent methods for extracting domain-invariant features from EEG and narrow down which techniques can potentially lend themselves to domain generalization.

### 2.1. Domain Adaptation

Domain adaptation constitutes a subset of transfer-learning techniques, which attempt to directly adjust learned model parameters to better accommodate a target dataset. The underlying principle is that data from two domains collected under different conditions can be assumed to have different feature distributions. The general goal of adaptation is to find a feature transformation where the domains are indistinguishable but still retain some of the original characteristics of the data [21]. In the case of EEG, it is often desirable to remove factors stemming from subjective characteristics while maintaining the effects of the phenomenon being studied (e.g., emotion). The majority of techniques currently being explored in this field are either kernel methods or DL methods.

#### 2.1.1. Kernel-Based Methods

Kernel methods are a class of algorithms that use kernel functions to project data onto a high-dimensional feature space where they are more likely to be linearly separable. These projections are typically calculated from inner product spaces, also known as Hilbert spaces, H, which also encode data similarity metrics. For more efficient computation, H is paired with a corresponding *reproducing kernel*, $k(x,x^{\prime })$, which when satisfying certain properties of linearity and continuity, defines a reproducing kernel Hilbert space (RKHS) [23]. This is the core of the *kernel trick*, which enables substitution of the kernel function, $k(x,x^{\prime })$, with a more efficient feature mapping, $<\Phi \left(x\right),\Phi \left(x^{\prime }\right)>$, where Φ is the feature map. This enables the kernel projection to be more efficiently applied to machine learning classification methods such as support vector machines.

Among domain adaptation kernel methods, one powerful kernel-based approach is proposed by transfer component analysis (TCA) [24]. TCA implements domain adaptation in RKHS by optimizing kernel parameters to minimize the maximum mean discrepancy (MMD) metric between domains. MMD essentially measures the difference between the means of the probability distributions of source and target feature mappings $\Phi (X_{S})$ and $\Phi (X_{T})$. As the distance between means approaches zero, the RKHS becomes more universal [22]. To preserve the original properties of the data, variance is maintained by maximizing the trace of the covariance matrix of the projected samples. However, an important limitation of TCA is that adaptation can only be applied to binary domains since the MMD metric is a pairwise calculation only suited for domain transfer.

A more recent work that builds upon TCA is maximum independence domain adaptation (MIDA) [25]. Similar to TCA, MIDA is an unsupervised domain adaptation technique that uses RKHS projection and combines it with a domain independence criterion. In this case domain independence is calculated using the Hilbert-Schmidt Independence Criterion (HSIC) [26], a non-parametric test statistic that is also effective as a kernel independence measure. MIDA works by first encoding the domain labels into “*domain features*” which are concatenated to the original features. Next, a nonlinear mapping is applied through a kernel matrix, $K_{x}=\Phi {\left(X\right)}^{T}\Phi \left(X\right)$, and the samples are projected to a subspace with dimension *h* using a learned projection matrix M. The final projection Z is equivalent to $W^{T}K_{x}$. Finally, domain adaptation is achieved by regularizing the kernel matrices $K_{x},K_{d}$ and projection matrix W by minimizing the HSIC. Additionally, a centering matrix adjusts the projected space to approximately zero mean. The main advantage of MIDA lies in the encoding of domain features into the projection, thus allowing the HSIC to work across multiple domains. A semi-supervised extension of MIDA (SMIDA) also allows for concurrent optimization of the mapping using target labels. Recent studies [22,27] have shown that MIDA outperforms other comparable methods like TCA and MMD in EEG-emotion datasets.

#### 2.1.2. Deep Learning Methods

In recent years, DL methods have steadily taken over the range of state-of-the-art of domain adaptation techniques [21]. In the context of EEG analysis, one of the first promising deep domain adaptation methods to be proposed was the Wasserstein generative adversarial network (WGAN) [28]. This method uses a pair of *generators*, neural networks trained to output a feature vector. A pretraining step initializes separate generators for the source and target domains. Both are set to output similarly sized vectors, therefore creating a shared feature space. Next, an adversarial training step adjusts the parameters of the generators such that the outputs more closely match each other based on a Wasserstein distance. Thereafter, the combined outputs of the adapted source and target feature spaces are used as input features for the output classifier network. Experiments on SEED and DEAP showed that the WGANDA framework showed significantly better results in subject-independent tests than those of the standard KPCA, TCA, and TPT approaches. However, the need for training different networks for each new domain makes it difficult to scale the technique to multiple domains. A separate generator would be necessary for each subject in the training set, increasing both memory and processing demands.

In another study that used a similar topography representation to our proposed work [14], domain adversarial networks (DANN) [13] were used to regularize intermediate feature embeddings for subject invariance. The proposed architecture featured separate feature embeddings for each hemisphere, as well as a combined embedding for the final whole brain. Each embedding was trained with a separate DANN, each of which was trained to recognize subject labels. By fine-tuning the embeddings adversarially to each target subject during testing, domain distribution differences could be minimized. As a result, the BiDANN model performed better than state-of-the-art approaches, including plain DANN and dynamic graph convolution networks [29], on the SEED dataset, and particularly in the subject-independent tests.

A more recent work [30] applied a similar hemispheric approach that also optimized for latent representation similarity between the source and target domains. In their proposed work, the architecture features source and target networks which are treated as separate models during training; however, they feature shared model weights. Shallow layer output features were adapted using marginal distribution adaptation through an adversarial domain predictor DANN. Meanwhile, the deep layers up until the final output features were adapted to the conditional distribution of the target label by combining the outputs of the source and target networks with association reinforcement. Results on SEED and DEAP show that their method is more effective than not only the baseline KPCA, TCA, TPT, but also recent neural network approaches like DANN, MMD and ADA. However, once again parameter fine-tuning simply adapts the distribution of the intermediate features to the target domain.

In many of these approaches, the model both identifies salient features from the source data and optimizes them to accommodate the target subject, in a binary domain transfer scheme. One key limitation of this scheme is that it requires retraining the model for each new subject. Systems designed for use by multiple users may require a model that shows good generalization across multiple subject domains. The base DANN method [13] is a notable exception since it is not necessarily limited to binary adversarial domains. In the following section, we discuss domain adaptation techniques that can be applied toward domain generalization.

### 2.2. Domain Generalization

Often with consumer products, it is necessary to have a system that is immediately usable by as many users as possible. This requires a domain agnostic or domain invariant model with reasonably good generalization performance. *Domain generalization* approaches achieve this by leveraging data from multiple sources to learn a universal representation that generalizes well across unseen target domains [31].

#### 2.2.1. Domain Agnostic Methods

In addition to methods that explicitly use domain labels, general-purpose techniques that are agnostic to the source/target domains can also be used for generalization. For instance, in traditional EEG analysis, better generalization is often achieved by using ICA [4] to decompose the signal into its source components and subsequently selecting only the most relevant components. Another typical use-case of ICA is for the removal of irrelevant noise effects from EMG or EoG. However, the need for manual intervention limits its applicability in automatic machine learning methods.

Fortunately, linear decomposition methods like PCA, which include inherent feature selection, have shown some promise for EEG domain generalization. Particularly, kernel PCA (KPCA) [32], has been shown to be somewhat effective for cross-domain classification [27]. While it is not specifically designed for domain adaptation, it is often used in comparisons to these types of methods [22].

KPCA is an extension to PCA that applies the kernel trick $K\left(x,y\right)=\Phi x^{T}\Phi \left(y\right)$ to project data non-linearly onto a kernel space where it may be more linearly separable. This space is a RKHS projection, similar to the one employed in MIDA. PCA is applied within this kernel space by computing the covariance matrix and determining the “*principal components*” of the projected data by ranking eigenvectors by their corresponding eigenvalues. By selecting a subset of eigenvectors which contribute most of the variance of the dataset and using them as basis for the final projection, a more generalized projection can be achieved. An important limitation of PCA is that it is sensitive to outliers since they may have a strong influence on the variance of the dataset.

Another study [33] proposed the use of variational mode decomposition (VMD) [34], a technique that combines signal decomposition and component selection, and combines it with DL. VMD is a time-frequency decomposition approach that algorithmically detects and decomposes a signal into its principal “*modes*”. This involves an iterative process where each mode is updated directly in Fourier domain using a narrow-band Wiener filter corresponding to the current estimate of the mode’s center-frequency. Meanwhile, the center frequency is re-estimated based on the center-of-gravity of the mode’s power spectrum. As a result, each intrinsic mode function (IMF) exhibits certain sparsity properties with regards to their bandwidths. That is, they have a limited bandwidth and are assumed to be compact around a center pulsation which is determined together with the decomposition. IMFs can serve the same purpose as principal components in PCA [6]; however, the former preserves time-frequency information by isolating the modes of the signal and can be used to generate a meaningful PSD. In Pandey and Seeja [33], the top three IMFs were selected and their PSD features were combined into feature vectors which were subsequently used to train a deep neural network (DNN). Through experiments on the DEAP dataset, they found that the VMD-based model performed better than comparable models trained on raw PSD or differential entropy features.

#### 2.2.2. Deep Latent Variable Methods

Another possible approach to domain generalization is to use neural networks to discover latent features in the data. This is comparable to the domain agnostic models, however DNNs also tend to scale better with larger datasets.

In a recent study [35], they proposed the use of variational autoencoders (VAE) to extract features for classifying EEG motor imagery. VAEs are generative machine learning structures that learn an n-dimensional embedding of data constrained to a distribution. An encoder network transforms data to an embedding space while a decoder network reconstructs the original input from the embedding. An additional constraint is applied to the distribution of the embeddings. By combining these objectives, the encoder is able to generate a well-distributed set of features. Their work shows that Gaussian VAEs can be used to effectively extract salient features from 2D EEG spectrographs, resulting in a model that outperforms the state-of-the-art subject-independent results for the BCI Competition IV 2b dataset. They also showed empirically that the ideal EEG features naturally follow a normal distribution based on a Kolmogorov-Smirnov test. We adopt a similar approach with VAEs but with an additional subject-invariant regularizer.

Alternatively, we can take a more unsupervised approach by allowing the machine learner to discover domain-invariant features. In Chai et al. [36], they proposed the use of autoencoders to enforce a consistency constraint, combined with subspace alignment techniques, to minimize domain differences. Their proposed subspace alignment auto-encoder (SAAE) uses MMD for domain-invariant regularization and showed competitive performance on the SEED dataset, outperforming baselines like TCA. A limitation of the work is that the subspace alignment requires data from all domains to be loaded onto memory simultaneously. This limits its scalability to multiple domains, so only binary domain transfer was explored.

#### 2.2.3. Multi-Domain Adaptation

To build generalized models, it may be necessary to consider marginal distributions across multiple domains. Fortunately, certain approaches used for domain adaptation exhibit characteristics that could be applied for domain generalization [27]. Notably, domain agnostic techniques and those that support multi-domain regularization can be used to enforce domain generalization. Among these, KPCA, VMD, and MIDA provide good comparative performance while offering varied approaches to generalization. Methods discussed in this section are summarized in Table 1.

### 2.3. Contributions of the Work

In summary, while recent advancements in DL have brought about marked improvements in EEG emotion recognition models, complex methods with many parameters still suffer due to the relatively small labeled datasets and the high subject variability of EEG. Topographic representations provide a practical middle ground that allows machine learning algorithms to discover high-level spatial-temporal context-specific features, while also reducing data requirements by utilizing manually extracted, but well-researched, effective low-level features (i.e., PSD). Meanwhile, domain adaptation has proven to be quite effective for handling subject variability; however, most methods require fine tuning of the model for each target subject. Contrarily, label-agnostic normalization methods do not cope well with small datasets with imbalanced feature distributions. For applications where subject-specific data are unavailable or model retraining is not computationally feasible, a flexible general-purpose model is needed.

The proposed work offers a flexible domain-generalized deep EEG emotion model that builds upon previous works by learning a bi-hemispheric variational embedding optimized for subject invariance. To work around the small-data constraints of EEG-emotion datasets, instead of building an end-to-end model, we use topographic inputs similar to Bashivan et al. [13] while also adopting the bi-hemispheric feature split of Li et al. [14]. This also enables better flexibility for combining trained models with other datasets in the future. To optimize for generalized feature embeddings that are not overfit to the training distributions, VAEs with Gaussian regularization similar to Dai et al. [35] are applied to the hemispheric topography features. These variational embeddings are further refined by combining them with domain adversarial training [37] across subjects to learn subject-independent feature embeddings. This increases the robustness of the proposed model to the effects of subject variability compared to purely distribution-driven methods like VMD and KPCA. Additionally, unlike domain adapatation methods like MIDA, the addition of VAE regularization should increase the resistance of our model to the overfitting effects of small datasets with non-Gaussian feature distributions. We validate the performance of the models by focusing on subject-independent classification performance when combined with a DL classifier, similar to the approach used in Pandey and Seeja [33]. Furthermore, we perform qualitative analysis of the embedding spaces using manifold learning algorithms to demonstrate the differences in the feature distributions.

## 3. Methodology

### 3.1. Input Data

**Figure 1:**
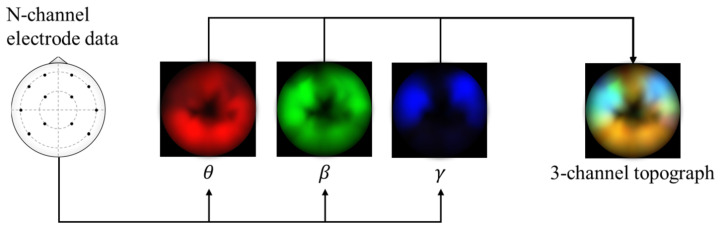
Alpha, beta, and gamma power are extracted from the electrode data, plotted onto separate topographic maps, and are combined into a 3-channel topograph used as input for the proposed model.

The framework in this study uses spectral topography data to maximize dataset inter-compatibility and to improve robustness to localized electrode noise. The pre-processing and topography generation were performed using MNE-Python [38] with the PSD calculated using the multitaper method [39] and bilinear spatial interpolation. For the target channel bands, we refer to findings from previous works [14,40,41,42] that have repeatedly shown that relatively high frequency bands typically lead to significantly improved emotion recognition performance. Therefore, we generate three 64 × 64 px topographs based on the following brainwave bands: alpha (8–13 Hz), beta (13–30 Hz), and gamma (30–50 Hz). Subsequently, these topographs are combined into a single 3-channel image with pixel values scaled between 0 and 255, as shown in Figure 1. This encoding has two main advantages: (1) it acts as a form of normalization to scale the values within a standard range, and (2) it allows the data to be visualized as an RGB image. This may be useful later for visualizing the DL model’s activations. These data are passed as input to a custom deep CNN.

### 3.2. Deep Learning Architecture

**Figure 2:**
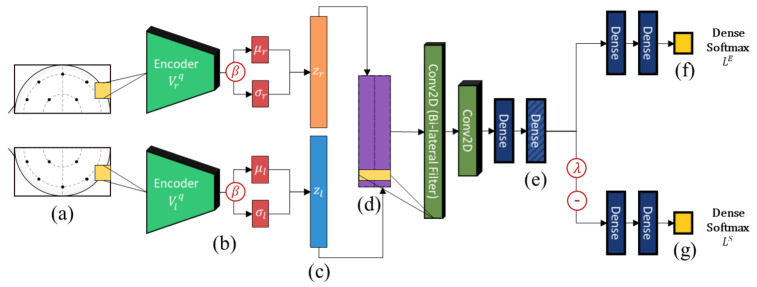
The high-level architecture diagram of the proposed model. From left-to-right, (**a**) EEG topograph inputs, (**b**) convolutional variational encoders, (**c**) lateralized embeddings, (**d**) bi-lateral feature concatenation and lateral convolution, (**e**) subject-independent dense embeddings, (**f**) emotion classifier, and (**g**) adversarial subject classifier.

An overview of the architecture of the proposed model is shown in Figure 2. Similar to Li et al. [14], we adopt a bi-lateral architecture to emulate the lateralized brain theories of emotion [43], which are frequently used in neuroscience and neuroimaging studies. In the context of machine learning, asymmetry features have regularly been found to improve results considerably when compared to just using electrodes separately [14,15]. This is enforced at the input level by cropping the topographs into left and right hemispheres each with their independent VAE weights. This ensures that feature embeddings from either hemisphere are properly regularized to a Gaussian distribution. The outputs of the lateral VAEs with β-scaled regularization (beta-VAE [44]) are then combined and filtered using a bi-lateral convolution to extract lateralized features. Thereafter, we impose generalization across domains through user adversarial regularization on the bi-lateral feature embedding by applying domain adversarial training. In this case, different subjects are treated as different domains. An adversarial classifier attempts to distinguish between different users, while applying a negative gradient to any upstream layers to train them to resist these attempts. Effectively, the model learns a feature embedding that is indiscriminate between users while jointly optimizing the features to distinguish the target emotion labels. Additionally, the Gaussian penalties of the VAEs prevent the model from converging to an unexpected out-of-bounds distribution that may also satisfy the adversarial and target classifiers.

### 3.3. Regularizing for Normally Distributed Features

The proposed model uses a pair of VAEs to generate the initial embeddings for the input EEG topographs. Aside from serving as a feature embedding regularizer, they have also been shown to be effective for generating new data and even a viable alternative to generative adversarial networks (GAN) [45]. Some studies [46,47] have also shown how VAE-based training can improve classification performance. It is hypothesized that VAE regularization leads to better-disentangled representations, which could explain the improved classification performance. In the context of EEG signals, Aznan et al. [48] demonstrated how VAE can be effective for simulating EEG samples which when used for pre-training models can improve the cross-subject accuracy. This is further supported by another recent work [35] that showed how CNN-VAEs can be effective for classifying motion imagery from EEG spectrograms.

In the proposed work, separate beta-VAEs are used for each hemisphere to allow the model to encode bilateral asymmetry features. We opt for the standard mean squared error (MSE) for the reconstruction loss in the VAEs despite common criticisms to MSE reconstructions when used in image classifiers. This is because the blurry reconstruction common to MSE-based VAEs is a non-issue for the EEG topograph inputs, which are inherently spatially interpolated.

#### Variational Autoencoders

**Figure 3:**
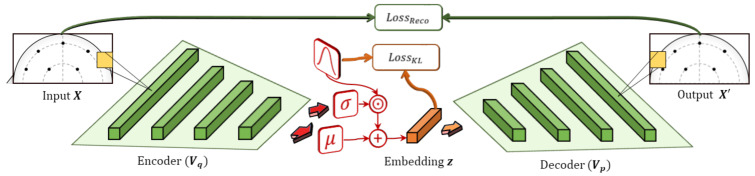
Variational autoencoders are composed of an encoder–decoder pair. The encoder outputs mean and s.d. components, which are used to sample the latent encoding, z, stochastically from a target distribution. It combines losses for the reconstruction of the input, x, and the KL-loss between the encoding, z, and the target distribution (i.e., Gaussian).

The VAE is a machine learning structure based on the autoencoder and includes both encoder and decoder components, as shown in Figure 3. The encoder learns to generate an embedding that is constrained in both number of dimensions and type of distribution. For instance, the embedding space can be configured to be an n-dimensional vector of normally-distributed values. The decoder then takes the embedding and attempts to generate an accurate reconstruction of the input. By combining these objectives with gradient optimization methods, the model learns an embedding that approximates latent factors inherent to the data, similar to matrix factorization. Additionally, the embeddings are constrained to a Gaussian distribution that allows the model to prioritize factors in the data that are well-distributed.

Originally formalized in Kingma and Welling [49], a VAE is a machine learning structure that is trained based on learned approximate inference and works by constraining the generated representations to some continuous distribution (e.g., Gaussian). VAEs provide a computationally efficient method to optimize deep latent-variable models jointly with inference models by solving the intractability problem of parameterizing the posterior distribution, $p_{\theta }\left(x|z\right)$, for the latent variables, z, given a marginal likelihood, $p_{\theta }\left(x\right)$ [50]. Similar to other variational methods, VAEs optimize for the *evidence lower bound* (ELBO) but also include a KL-divergence term, redefining the ELBO loss as

(1) $$L_{\theta ,\phi }\left(x\right)=\log p_{\theta }\left(x\right)-D_{\mathrm{KL}}\left(q_{\phi }\left(z|x\right)\right)\left|\right|p_{\theta }\left(z|x\right).$$

The KL term indicates the KL divergence of the approximate posterior, $q_{\phi }\left(z|x\right)$, from the true posterior, $p_{\theta }\left(z|x\right)$. Due to the non-negative property of the KL term, the ELBO loss acts as a lower bound on the log-likelihood of the data. Here, the VAE employs a parametric *inference model*, $q_{\phi }\left(z|x\right)$, also known as the *encoder*, which is parameterized by θ to approximate the intractable posterior, $p_{\theta }\left(z|x\right)$. By approximating the posterior with a generative model, $p_{\theta }\left(x,z\right)$, the ELBO loss can be applied to enable the joint optimization of parameters θ and ϕ, using stochastic gradient descent, as defined in the AEVB algorithm [49].

Thus, if we assume that $p_{\theta }\left(z\right)=N\left(z;0,I\right)$ and that the true posterior for our latent encoding approximates to a Gaussian distribution, then the approximate posterior becomes a multivariate Gaussian defined as

(2) $$\log q_{\phi }\left(z|x_{i}\right)=\log N\left(z;\mu_{i},\sigma_{i}^{2}I\right),$$

where $\mu_{i}$ and $\sigma_{i}$ are the mean and s.d. outputs of the encoder for input $x_{i}$, parameterized by ϕ. To calculate the gradients through stochastic sampling, the *reparameterization trick* is applied by sampling from the posterior, $z_{il}∼q_{\phi }\left(z|x_{i}\right)$, with $z_{il}=\mu_{i}+\sigma_{i}⨀\epsilon_{l}$, where $\epsilon_{l}∼N\left(0,I\right)$ and ⨀ denotes an element-wise product. Thus, the resulting loss equation for the Gaussian implementation of VAE can be defined as

(3) $$L_{VAE}\left(\theta ,\phi ;x_{i}\right)≃\frac{1}{2}\sum_{j=1}^{J}\left(1+\log\left({\left(\sigma_{ij}\right)}^{2}\right)-{\left(\mu_{ij}\right)}^{2}-\sigma_{ij}^{2}+\frac{1}{L}\sum_{l=1}^{L}\log p_{\theta }\left(x_{i}|z_{il}\right)\right),$$

where *J* is the feature dimension of the latent embedding, z, and *L* denotes the number of random samples taken (typically, L=1).

Our proposed model implements beta-VAE [44], an extension to the base VAE that introduces a controlled parameter, β, that allows tuning of the learning constraints of the model. Specifically, it augments the KL-divergence term in Equation (1) into $\beta D_{KL}(q_{\phi }\left(z|x\right)||p\left(z\right))$. This enables stronger disentanglement between latent factors by increasing the regularization loss and also allows the adjustment of the regularization strength during training. Recent investigations have found that scheduling β to increase linearly during training results in improved model convergence [51]; therefore, we adopt a similar training procedure.

Optimizing these objectives enforces improved sampling of latent features despite biases in the training distribution. Non–Gaussian distributions are often attributed to insufficient data or problems in the data sampling methodology. In effect, they do not generalize well. By constraining the reconstruction objective to finding Gaussian embeddings, the model discovers features that are better suited for generalization. That is, the model can ignore outliers or badly sampled factors and biases inherent to the training data to some extent since it models the distribution of clean data [52]. A model trained on these losses learns a stochastic mapping from the x-space defined by the available training dataset, to a latent z-space embedding regularized to a distribution that more closely matches what we would expect from a more comprehensive dataset (i.e., Gaussian). When used as a pretraining method, such regularization has been found to improve the classification performance by learning factors of variation that improve data log likelihood [51]. This is particularly useful in our case since the high-dimensional nature of our dataset combined with the relatively small sample size increases the possibility of overfitting to the training distribution. Without proper regularization, there is a high probability that standard deep learning models will develop a strong bias to the training set resulting in poor performance on unseen data.

**Figure 4:**
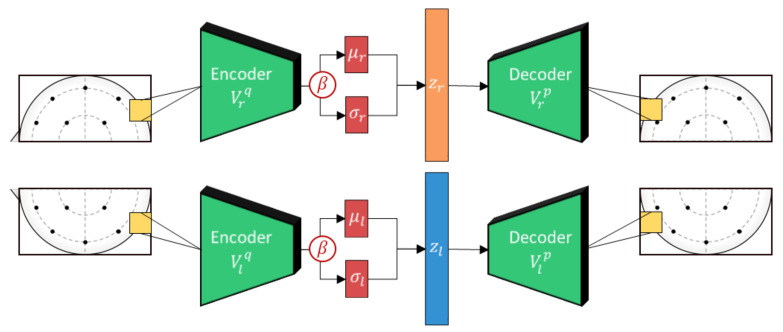
The lateralized beta-VAE implementation used in this work. Each hemisphere is composed of an encoder and decoder pair with the encoder generating a stochastically sampled embedding, *z*, based on outputs, μ and σ. Separate weights are used to encode the features for each of the hemispheres to adhere to neuroscience theories of lateralization.

Next, the hemispheric VAE outputs are vertically concatenated and processed with a bi-lateral convolutional filter to extract lateral brain activity features as shown in Figure 4. This is inspired by the lateral brain theories of emotion [43] and is an approach that has proven effective in previous works [13,14]. However, unlike in previous works, the VAE regularization should force the bi-lateral feature embeddings to be well-distributed, emulating distributions expected from large comprehensive datasets. In addition, to further curtail the effects from subject variability, we apply subject-independent regularization using domain adversarial training.

### 3.4. Optimizing for Subject-Independence

**Figure 5:**
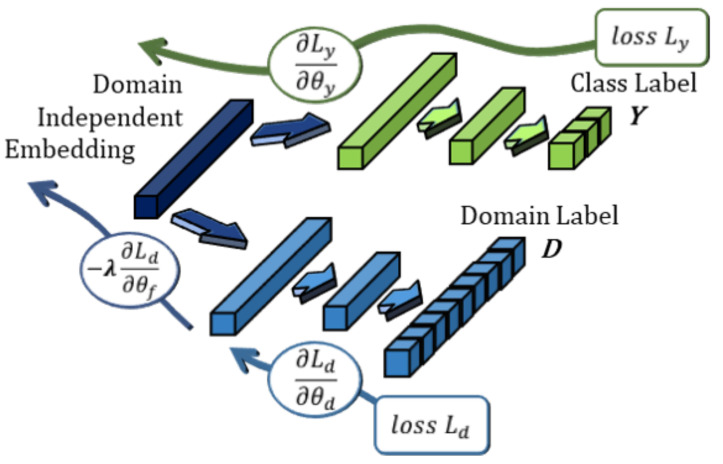
The domain adversarial network (DANN) implementation used in this work. The feature embedding layer is shared by the emotion classifier and the adversarial subject domain classifier. Each classifier is trained to classify for its respective task; however, the gradient is reversed and scaled with parameter λ, in effect training upstream layers for subject-independence.

To achieve better generalization across different subjects, we regularize the bi-lateral filter features with DANN training. DANN is a domain alignment approach usually presented in domain adaptation studies as an alternative to GANs [19]. The underlying theory is that effective domain transfer can be achieved by finding features that are indiscriminate between the training (source) and test (target) domains [37]. While it is primarily used for adapting between binary source/target domains, in this work we apply it together with the features extracted from the VAE embeddings to enforce subject-invariance. A similar approach for speaker recognition is found in [53] where it showed promising results for generalizing across different audio datasets. In this case, we treat each subject as an independent domain that is assumed to be sampled from a different distribution and apply the regularization starting from a feature embedding layer shared by the emotion classifier and the adversarial classifier, as shown in Figure 5.

#### Domain Adversarial Neural Networks

One of the earliest works formalizing DANN adaptation [37] tested the technique on optical character recognition (OCR) models by adapting pretrained models to different OCR datasets. As a general-purpose architecture, it is defined primarily by its joint optimization of a *prediction loss* and a *domain loss*. In a simplified case featuring a single hidden layer DANN, the prediction and domain losses can be defined by the following:

(4) $$\begin{array}{c} L_{y}^{i}\left(\theta_{f},\theta_{y}\right)=L_{y}\left(G_{y}\left(G_{f}\left(x_{i},\theta_{f}\right);\theta_{y}\right),y_{i}\right),\\ L_{y}^{i}\left(\theta_{f},\theta_{d}\right)=L_{d}\left(G_{d}\left(G_{f}\left(x_{i},\theta_{f}\right);\theta_{d}\right),d_{i}\right) \end{array}$$

Here, $G_{f}$ is defined as the neural network feature extractor, $G_{y}$ is the part of the neural network responsible for label classification, and $G_{d}$ is the neural network domain classifier. The parameters, $\theta_{f},\theta_{y},\theta_{d}$, refer to the trainable parameters of their corresponding neural networks. Training the DANN consists of finding the saddle points for parameters ${\hat{\theta }}_{f},{\hat{\theta }}_{y},{\hat{\theta }}_{d}$, such that

(5) $$\begin{array}{c} {\hat{\theta }}_{f},{\hat{\theta }}_{y}=\underset{\theta_{f},\theta_{y}}{arg min}E\left(\theta_{f},\theta_{y},{\hat{\theta }}_{d}\right),\\ {\hat{\theta }}_{d}=\underset{\theta_{d}}{arg max}E\left({\hat{\theta }}_{f},{\hat{\theta }}_{y},\theta_{d}\right) \end{array}$$

As is apparent from the parametric saddle point in Equation (5), the objective for ${\hat{\theta }}_{d}$ runs adversarial to that of the other classifiers. Instead of minimizing the error, it is necessary to maximize it; therefore, the combined objective for the feature generator can be defined simply as

(6) $$L_{DANN}\left(\theta_{f},\theta_{y},\theta_{d}\right)=L_{y}^{i}\left(\theta_{f},\theta_{y}\right)-\lambda \left(L_{d}^{i}\left(\theta_{f},\theta_{d}\right)\right),$$

where λ is a scaling factor that controls the strength of the reversed adversarial gradient. Typically, λ=1; however, this hyper-parameter can be tuned if either the target classifier cannot reach convergence or the feature embedding is not sufficiently domain-invariant. Empirical testing has also shown that introducing an unsupervised pre-training step as described in Algorithm 1 leads to better convergence.
**Algorithm 1:** Bi-lateral VAE Pre-training
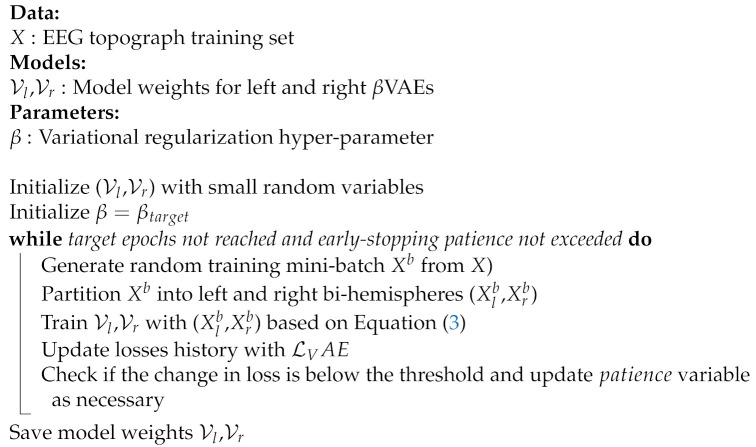


### 3.5. Training Phases

#### 3.5.1. Unsupervised VAE Pretraining

Before training the full model, it may be beneficial to pretrain the VAEs in an unsupervised manner with the training set, as shown in Algorithm 1. This step reduces the risk of exploding error gradients from the large losses during the initial phases of jointly training the VAEs and the classifiers. We also found that this practice increases the convergence rate of the subsequent supervised and semi-supervised training phases by providing a reusable starting point for supervised training.

#### 3.5.2. Supervised EEG-BiVDANN Training

The proposed model is trained to optimize for generating feature embeddings that are indiscriminate across subjects in the training set. This is hypothesized to also approximate toward indiscriminate features for unseen test sets from the same general sampling population. To optimize for both the variational loss and the adversarial loss, our model uses interleaved adversarial training, VAE reconstruction, and classifier optimization, as described in Algorithm 2.
**Algorithm 2:** Bi-lateral VAE Pretraining
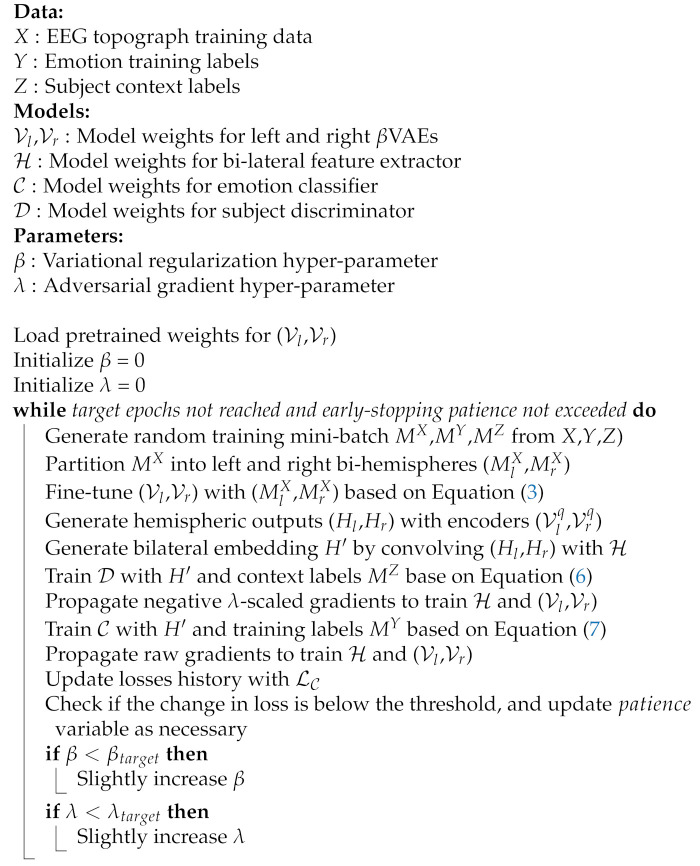


A jointly optimized model was also tested, however it was found that the interleaved training allows for finer control and tuning of the individual regularizers. Properly tuning the regularization strengths is essential for preventing the independent losses from overwhelming each other, particularly with the target label classifier.

#### 3.5.3. EEG-BiVDANN Model Objective

To build a subject-generalized model of EEG emotion, our proposed BiVDANN adopts the joint optimization of three losses: (1) VAE loss (Equation (3)), (2) subject-independent DANN loss (Equation (6)), and (3) emotion classifier loss $L_{C}\left(\phi_{v},\theta_{v},\theta_{f},\theta_{y}\right)$. Formally, the combined loss can be defined as

(7) $$L_{BiVDANN}\left(\phi_{v},\theta_{v},\theta_{f},\theta_{y},\theta_{d}\right)=L_{\beta VAE}^{l}\left(\theta_{v}^{1},\phi_{v}^{l}\right)+L_{\beta VAE}^{r}\left(\theta_{v}^{r},\phi_{v}^{r}\right)+L_{y}\left(\phi_{v},\theta_{f},\theta_{y}\right)-\lambda \left(L_{y}\left(\phi_{v},\theta_{f},\theta_{d}\right)\right)$$

The adversarial minimization and maximization objectives are thus defined as follows:

(8) $$\min_{\phi_{v},\theta_{v},\theta_{f},\theta_{y}}\max_{\theta_{d}}L_{BiVDANN}\left(\phi_{v},\theta_{v},\theta_{f},\theta_{y},\theta_{d}\right)$$

Internally, Equation (8) encapsulates the following min-max optimization steps:

(9) $$\begin{array}{cc} {\hat{\phi }}_{v},{\hat{\theta }}_{v} & =\underset{\phi_{v},\theta_{v}}{arg min}L_{BiVDANN}\left(\phi_{v},\theta_{v},{\hat{\theta }}_{f},{\hat{\theta }}_{y},{\hat{\theta }}_{d}\right),\\ {\hat{\phi }}_{v},{\hat{\theta }}_{v},{\hat{\theta }}_{f},{\hat{\theta }}_{y} & =\underset{\phi_{v},\theta_{v},\theta_{f},\theta_{y}}{arg min}L_{BiVDANN}\left(\phi_{v},\theta_{v},\theta_{f},\theta_{y},{\hat{\theta }}_{d}\right),\\ {\hat{\theta }}_{d} & =\underset{\theta_{d}}{arg max}L_{BiVDANN}\left({\hat{\phi }}_{v},{\hat{\theta }}_{v},{\hat{\theta }}_{f},{\hat{\theta }}_{y},\theta_{d}\right), \end{array}$$

where the saddle point is defined by symbols ${\hat{\phi }}_{v},{\hat{\theta }}_{v},{\hat{\theta }}_{f},{\hat{\theta }}_{y},$ and ${\hat{\theta }}_{d}$. When these symbols are found on the right-hand side of Equation (9), it indicates that their respective parameters are not optimized by the current operation. Tuning of the models relies primarily on selecting hyper-parameter values that balance the main optimization objectives and may vary based on the dataset.

### 3.6. Datasets

In this study, we use two of the largest and most widely studied EEG-emotion datasets: DEAP [54] and SEED [15]. Both datasets feature multimodal data; however, we constrain this study to only the EEG portion of the data. Similarly, both also include various types of annotations; however, we focus on the ones most relevant to emotion.

For DEAP, the dataset comprises data from 32 subjects, each with a single continuous data gathering session divided into 40 trails that last about 1 min each. Emotion was elicited using music videos and labels were collected with a questionnaire including a self-assessment manekin (SAM) for arousal and valence (9-point scale). The EEG device used for measurement was a 32-channel Biosemi headset capable of recording at a 512 Hz data rate; however, the final cleaned data were downsampled to 128 Hz. Since we focus only on the emotion classification aspect, we train the models to classify a binary valence where valence annotations >5 are *positive*, and those <5 are *negative*. The selection of the valence threshold in other works tends to be rather arbitrary; however, here we opt to use 5 as it is the median value of the SAM questionnaire.

For the SEED dataset, it comprises data from 15 subjects, with each having participated in 3 separate sessions divided into 15 trials, which were approximately 4 min long. Emotions were elicited using emotional film clips pre-labeled as positive, neutral, or negative. For the EEG device, they used a 62-channel ESI Neuroscan system capable of recording data at 1000 Hz but with the final stored data downsampled to 200 Hz.

### 3.7. Data Preparation

To generate the representative topographs from the raw EEG data, we apply a sliding window method. For this, we consider that labels in both datasets are applied across entire trials. Thus, to increase the probability that most segments included the target labeled emotions we employ a 15 s sliding window with a 50% overlap. This is roughly based on the findings of a previous study [55] that found ∼12 s to be optimal for valence classification. To extract the time-frequency features and generate the topographic representations we use MNE-Python [56], an open-source package for MEG and EEG analysis. Specifically, we apply discrete prolate spheroidal sequences (DPSS) tapers to extract the PSD spectrum [57]. This method avoids the spectral leakage that is common in methods that use the standard Welch periodogram. A 64 × 64 px topograph is generated for each brainwave band of interest (alpha, beta, and gamma), resulting in three-channel matrix whose values are then scaled between 0 and 255 to generate the input topograph image.

### 3.8. Model Implementation

The bulk of the machine learning components in this work are implemented in Python, specifically using the Keras machine learning API [58]. All models were optimized using the Adam optimizer [59] since it could best handle the highly variable gradients produced by the joint optimization. For the purpose of discussion, we will divide the proposed BiVDANN into its main components: bi-lateral feature extractor, emotion classifier, and adversarial subject classifier. A description of each is shown in Table 2.

## 4. Experiments and Results

### 4.1. Training Hyperparameters

The proposed model jointly optimizes the multiple objectives specified in Equation (7); however, properly controlling conflicting gradients requires fine-tuning of various regularization weights, particularly β and λ. Empirical tests show that linearly increasing both β and λ during the early stages of training allows the model to converge to a distribution with the desired characteristics. Specifically, we grow β from zero to full magnitude during the first 100 epochs, while λ had a slightly faster increase reaching max in the first 50 epochs. Testing showed that this configuration prevents the model from starving the target emotion learning objective. Slowly growing beta also prevents the gradient from exploding due to changes in KL-loss while the model is also exploring the main class objective during the initial learning phase. Similarly, slowly increasing lambda allows the model to first learn a strong user discriminator that will later serve as a powerful regularizer when the negative adversarial gradient reaches a critical point where it matches or overpowers the class gradient. For the training epochs, a batch size of 256 samples was used to allow the adversarial batches to contain a good distribution of subject samples, and empirical testing showed that 300 epochs allowed all models to converge to an optimum state with the help of early stopping. We used 15% of the training data (∼1200 samples) for validation and set the early stopping objective based on the emotion class validation accuracy.

**Figure 6:**
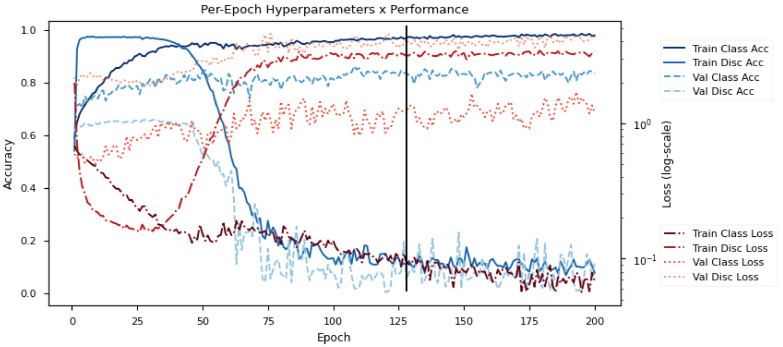
Sample training progression for DEAP data. The graphs indicate the training/validation accuracy/losses for the emotion classifier/subject discriminator over training epochs. The black line indicates the point used for early stopping.

Figure 6 shows the learning progression of the model on DEAP. Specifically, it shows the testing losses and accuracies (*Loss/Acc*) on the training and validation sets (*Train/Val*) for the emotion classifier and the subject discriminator (*Class/Disc*) over the epochs. Here, the model is learning with 2 emotion classes (*Positive*/*Negative*) and 32 subject classes. The objective of the model is to first learn a good subject discriminator, and then to optimize the emotion classifier with features that minimize the performance of the subject discriminator. The use of adversarial objectives pushes the emotion classifier toward subject-invariant features.

The early training phase is characterized by a rapid decrease in all losses (red lines), particularly the discriminator loss. This demonstrates the strong influence of subject variability on the dataset since the subject discriminator learns much faster than the emotion classifier despite having more subject classes (32 in the case of DEAP). If allowed to continue, there would be an eventual increase in the classification losses due to subjective overfitting. However, as λ reaches a critical point at around epoch 40, the adversarial gradients begin to tune the embedding for subject invariability. Eventually, this converges to a representation that is indiscriminate across subjects but is highly discriminative for emotion classes.

### 4.2. Comparison Results

For the comparison experiments all the featured methods used a similar *Emotion Classifier* component indicated in Table 2. This allows the comparison to be more even; thus, we can focus on comparing the efficacy of the different feature embeddings. Specifically, the following techniques are used for comparison:

- *CNN*—simple 2D CNN featuring 3 × [Conv2D(3 × 3) + BatchNorm] layers with ∼200,000 trainable parameters
- *Resnet-50* [60]—popular DL network for image classification with ∼23 million trainable parameters
- *KPCA* [61]—nonlinear dimensionality reduction technique
- *VMD* [34]—signal decomposition technique used for EEG analysis in Pandey and Seeja [33]
- *MIDA* [25]—semi-supervised domain adaptation technique for mapping data to domain-invariant subspace

In this experiment, we wish to compare the generalization ability of the models. Therefore, we apply leave-one-subject-out cross-validation for all the quantitative experiments. That is, the entire dataset is divided such that all samples from a single subject are excluded from training a model. Subsequently, all samples from that subject are used to measure the test performance of the trained model. This is performed for each subject, and we use the mean accuracy and standard deviation to evaluate the models.

We wish to compare our proposed approach to the standard baseline methods, as well as to the other comparable domain generalization techniques. For the standard baselines, we use a standard shallow CNN to represent DL models with lower data requirements, and ResNet-50 [60] to assess the performance of more complex DNNs. These are both single-objective models that aim only to minimize the error on the training set. As such, they can be used as an indication of the inclination of the datasets towards overfitting to the training distribution. We can expect most classic DNNs applied to the same data to exhibit similar limitations.

For the generalization approaches, we combine feature extraction procedures that optimize for subject generalization. Since there are currently few state-of-the-art generalization techniques applied to EEG that experience improvements without including samples from the test domain, we use label-agnostic and multi-label modeling techniques for comparison. The algorithms compared include KPCA [61] to represent nonlinear label-agnostic dimensionality reduction approaches, VMD [34] for the EEG signal decomposition approach, and MIDA [25] for multi-domain adaptation. For VMD, we apply feature extraction on the raw EEG similar to [33], although we apply the same 15 s window used in the other methods. The results are shown in Table 3 and Table 4 below.

Label-agnostic approaches such as KPCA and VMD operate solely based on the distribution of the data they are modeling. For KPCA, a nonlinear mapping is generated based on the principal components of the data and how much each contributes to the overall variance. Thus, they are suitable for identifying strong latent factors. However, this also makes them vulnerable to noisy data and outliers. Conversely, VMD is a signal decomposition method that is designed to be resistant to noise effects, particularly those in nonlinear and non-stationary signals. It decomposes a signal into a finite number of IMFs which are comparable to KPCA’s principal components. Moreover, the sifting process generally leads to approximately Gaussian distributed modes which can minimize the effect of outlier noise. These two algorithms can indicate how techniques driven only by data distribution perform on EEG datasets. They can also indicate the strong impact of the signal noise and outliers on the datasets, since they lie on opposite sides of the spectrum with regards to this.

Near the end of the section, we also include model ablation tests to investigate the performance of basic VAEs and DANNs on these datasets.

Table 3 shows the performance on the SEED dataset. Here, the models were classifying three different emotion classes (positive, negative, neutral);thus, the random point lies at ∼33%. All models could classify significantly better than random; however, our proposed BiVDANN achieved the best overall subject-independent performance. The MIDA-DNN model also achieved high relative accuracy, displaying the advantage of enforcing subject-invariance for generalized models. In contrast, the shallow CNN got the lowest result likely due to overfitting. This is also reflected by Resnet-50, which only exhibits marginally higher performance than the shallow CNN despite having a much higher number of parameters.

Next, Table 4 shows the results on the DEAP dataset. Despite being only a binary classification problem, all the models had greater difficulty with this task compared to the 3-class SEED dataset case. Once again, our BiVDANN achieved the best overall accuracy, followed by MIDA. Similarly, the CNN and Resnet-50 models fared the worst, this time showing more similar performances. However, this time, their performance was not significantly better (*p* > 0.05) than that of a random classifier. This indicates that without proper controls, models trained on the DEAP dataset may have a higher probability of leaning toward overfitting. One possible cause of this could be that each subject only contributes a single continuous session of data. This may have lead to a compounding of environmental and subject-induced variances. By environmental variances, we refer to variabilities such as how the headset was worn, the background EM noise at the time of recording, and other external sources that could affect the signal. In contrast, the multiple sessions in the SEED dataset may be reducing the overfitting effect of these environmental variances since each subject’s training data is composed of combined data distributions across different sessions. However, more study is required to confirm this hypothesis.

### 4.3. Qualitative Analysis of Embeddings

Next, we wish to evaluate the effects of the characteristics of the different embeddings generated by the different methods on the model performance. To visualize the embeddings, we apply t-Distributed Stochastic Neighbor Embedding (t-SNE) [62] and multidimensional scaling (MDS) [63]. t-SNE is a nonlinear dimensionality reduction method that focuses on preserving small pairwise distances or local similarities between neighbors. Conversely, MDS is a technique for plotting data in lower dimensions while preserving a global distance metric (e.g., Euclidean distance). It works by using pairwise dissimilarities to reconstruct a mapping that preserves the triangle distances. t-SNE is effective at emphasizing data clustering, while MDS is more effective at showing the global patterns of the data distribution. Here, we use the 2D maps to qualitatively assess how the data points and classes are clustered in the embedding spaces for the different techniques. To generate the visualizations, we train the respective algorithms with a random 70% batch from the datasets. As for the inputs to the visualization algorithms, in the case of the deep models, we use the *embedding layer outputs* of the models, or in other words, the data embeddings positioned right before the final set of fully-connected classification layers, as shown in the right side of Figure 2. For VMD and MIDA, we use the raw features generated by the algorithms. Finally, for KPCA, we visualize the embeddings using the first two principal components.

#### 4.3.1. SEED Embeddings

From the embeddings of the SEED dataset shown in Table 5, we observe that all the DL models can generate features that are clearly optimized for emotion separation. However, for the CNN and Resnet-50 models, we also observe some subject-based clusters leaking into the distributions. This indicates the presence of strong subject-dependent effects in the dataset and could indicate that features found by these models are also somewhat subject-dependent. Judging by their weak classification performance in the subject-independent tests, this inherent dependence may significantly limit their generalization performance. Meanwhile, for KPCA, we also observe some class-based separation of clusters, although subject clusters are much more dominant here than in the other models. This indicates that the variances of the features themselves are strongly influenced by the different subjects. This typically leads to poor generalization performance, as indicated in Table 3. VMD seems to be an exception to this since it shows heavy subjective clustering effects despite exhibiting better performance than the other baseline models. This could be due to its relatively normal distribution of features as shown in the MDS visualization. Meanwhile, for MIDA, the HSIC-based regularization exhibits the best mixing of the subject clusters, however this comes at the expense of the class clusters which show many incorrectly mapped data points. Finally, for the BiVDANN embeddings, we see strong class separation and minimal subjective influence in the feature distributions. The model is also able to generate a much more Gaussian feature distribution that emulates a more comprehensive dataset while prioritizing class separation.

#### 4.3.2. DEAP Embeddings

Next, we analyze the embeddings for the DEAP dataset shown in Table 6. Again, we observe a similar pattern in the case of the DL models, where the embeddings show clear emotion class separation. However, there is also much stronger subject-dependent clustering compared to the results from SEED. This is most evident for the CNN model, where subject clusters are even more dominant than the class clusters. This could explain the poor performance of the CNN model, which was only slight better than a random classifier at just 51.64% binary accuracy. Resnet-50 was better able to avoid subject-dependent effects; however, it also generated separate and lopsided clusters for the negative and positive classes. In this case, most of the testing samples are seen between the two clusters, leading to poor classification performance. The results for KPCA are once again similar to SEED, where subject-based clusters dominate the feature distribution. Therefore, it is unsurprising that its classification performance was similarly poor. Meanwhile, the BiVDANN was once again able to generate a feature distribution that separates the emotion classes while minimizing overfitting to subject-based variances.

#### 4.3.3. General Observations

Based on the visualization results, we made several observations. First, we see that while class-based clustering is generally favorable, the generalization performance is also strongly influenced by other effects, such as subjective clustering. It may be better to minimize subjective clustering, particularly for datasets where distributions naturally lean towards subjective variance, as shown by the KPCA tests. Second, even in cases where subjective clustering is avoided, other unlabeled variances can also contribute to distribution imbalances to reduce generalization performance, such as in the Resnet-50 embeddings. Conversely, the VMD results demonstrate that well-distributed embeddings, even when strongly influenced by subjective variances, can still produce well-performing models. In summary, the best results seem to come from embeddings that favor class-based clustering and avoid subjective variances, while maintaining a balanced (i.e., Normal) data distribution.

With the qualitative analysis, we showed that our proposed model successfully achieved its main objectives of generating an effective generalized feature embedding for EEG emotion classification. Unlike label-agnostic approaches, such as KPCA, it can leverage subject label data to avoid the effects of subjective variance. Additionally, unlike single-objective methods like unregularized DL techniques, it generates a more contiguous distribution, which seems to better accommodate unseen data samples. This is best demonstrated in Table 6, where both CNN and Resnet-50 generate good class-based clusters, but also have distributions with a high tendency of test samples landing between or outside of the known clusters. This could explain the poor classification performance and showcases the unpredictability introduced when treating emotion as discrete classes. Intuitively, negative and positive emotions should not be clearly separable classes; rather, they are different ends of the same spectrum. In effect, we expect that their distributions should overlap possibly at the point where neutral emotions should be located. By generating models that violate this smooth continuity, we may end up with overfitting representations that perform poorly on new samples. This is generally avoided by employing large comprehensive datasets. However, in cases where such a dataset is not available, such as in EEG-emotion analysis, regularization constraints, such as in our proposed BiVDANN, can provide a good alternative. In the next section, we test the contributions of the different components of BiVDANN with model ablation tests.

### 4.4. Model Ablation Study

For the model ablation study, we are interested in learning the contribution of the different components of the system to the overall model performance. The BiVDANN model features the following main components: bilateral inputs, variational regularizer, domain-independent regularizer, and emotion classifier. To test each of these components independently, the following models are trained and tested:

- *BiCNN*—includes bilateral inputs, but no variational regularization or domain- independent regularization.
- *BiVAE*—includes bilateral inputs, includes variational regularization, but no domain-independent regularization.
- *BiDANN*—includes bilateral inputs, includes domain-independent regularization, but no variational regularization.
- *L-VDANN*—only left-hemisphere inputs, includes both variational regularization and domain-independent regularization.
- *R-VDANN*—only right-hemisphere inputs, includes both variational regularization and domain-independent regularization.

The subject-independent test results for the ablation tests are summarized in Table 7 and Table 8. The tests show that without the regularizers, the performance of the BiCNN model is equivalent to that of Resnet-50. As such, we can expect other complex unregularized deep learning models to show similarly overfit performance. Next, the BiVAE model shows improved results with the aid of variational regularization. By jointly optimizing for emotion classes and a normal distribution, it achieves better performance than the VMD model, but worse than the MIDA model. The BiDANN model shows mostly equivalent performance to the MIDA, indicating that the domain-independent regularization is roughly equivalent to MIDA. Compared to BiVAE, BiDANN also shows comparable though slightly weaker performance. For the unilateral input tests, both models perform better than most other ablation models except for the BiDANN. This showcases the generalization ability of models that utilize the DANN regularization. Additionally, the R-VDANN model shows better performance than the L-VDANN model, though neither performs better than the bi-lateral BiVDANN. These results support the bilateral theory of emotion, although it also lends some credence to the right hemisphere hypothesis in the case of the unilateral input models.

## 5. Discussion

Among generalized models that do not require additional fine-tuning, BiVDANN showed superior performance, beating comparable state-of-the-art techniques including KPCA, VMD and MIDA, even when these were supplemented with DNN classifiers. It consistently achieved the highest subject-independent accuracy on both the SEED and DEAP datasets and showed promising performance even when constrained to only right hemisphere features. The qualitative embedding analysis also revealed clues regarding the advantages of the proposed model. Unlike label-agnostic approaches, such as VMD and KPCA, BiVDANN directly addresses the primary source of signal noise, which is subject variability. This results in features that are less prone to overfitting to certain subjects. Meanwhile, compared to purely context-focused approaches, such as DANN and MIDA, the proposed model can discover a well-distributed Gaussian feature space even when given a relatively small training set for the large number of model parameters. The VAE pretraining step also allows the model to learn salient features even when emotion labels are not yet available. As more EEG data become publicly available, it will be interesting to see at what point databases can achieve generalized distributions by virtue of their sample sizes. At that point, Gaussian regularization may no longer be necessary and regularization can be geared more toward other sources of variability.

Ablation testing allowed us to assess how each component of the model contributed to the improved performance. Among the ablated models, subject adversarial training (BiDANN) showed the best overall improvement. This supports the initial hypothesis that subject variability is a primary limiting factor for EEG-emotion models. Meanwhile, the VAE regularized model seemed to allow unseen samples to better fit within the primary distribution as opposed to the periphery, which may be overfit to a single emotion label. With non-regularized distributions, there is an increased chance that unseen users form their own feature clusters regardless of the emotion label. Another interesting finding was that a right-hemisphere model outperformed the left-hemisphere model, lending itself to the right-hemisphere hypothesis of emotion. Nevertheless, neither model could outperform the bi-lateral model; thus, lateralization may still play an important role in detecting human emotion.

Overall, it appears that the proposed model achieves its goal of providing a good generalized representation without the need for retraining. However, this comes with a trade-off in overall peak performance which was well below that of fine-tuned models adjusted for the target subject’s data [14,28,30]. Among these works, some [28] have claimed over 90% accuracy under certain conditions; however, it is unclear what affects this and how much manual tuning is required to get these results consistently for each new subject. It is also uncertain if this is a reasonable expectation across datasets when considering the inherent unpredictability of human emotions and the dataset labels themselves. Investigating this could be a challenge for future works and may be essential to proving if machine learning models are actually detecting emotion or if there is some other neurological phenomenon guiding the models. In any case, our approach provides a good starting point for general-use EEG-based systems while reserving the possibility for additional fine tuning when the necessary data requirements for new subjects are met.

One issue that remained unaddressed is the influence of EMG artifacts, particularly from facial expressions. This is a problem that is rarely addressed even in the related literature. EEG preprocessing in most publicly available EEG-Emotion datasets is often limited to bandpass frequency filters applied by their respective authors (4–45 Hz for DEAP and 0–75 Hz for SEED). While some datasets provide EMG readings, this is not a standard followed by most. Advanced source separation and decomposition methods remain good options for removing these artifacts, given enough time and expertise. However, in the interest of providing a scalable and easy-to-use method, these were not used in the current work. As with most machine learning methods, if these artifacts prove to be discriminative of emotion, they will be prioritized by the algorithm. More research may be needed to ascertain that machine-learned models are in fact using brain-sourced signals for detecting emotions. This may involve proving that learned features are sufficiently decorrelated from facial movements.

## 6. Conclusions

In this paper, we propose the BiVDANN model, which learns a generalized model of emotion by jointly optimizing objectives for learning normally distributed and subject-independent feature embeddings. It uses spectral topography data as the input to maximize dataset inter-compatibility, improve robustness to localized electrode noise, and enable investigations into neuroscience theories, such as the neural lateralization of emotion. The topography maps are processed by layered convolutions connected to a pair of beta-VAEs with Gaussian regularization. This allows the model to generate generalized hemispheric feature embeddings from relatively small datasets with biased feature distributions. This is followed by bi-lateral convolutions combined with domain adversarial training to discover subject-independent features. Thus, the resulting BiVDANN model learns a feature embedding that effectively separates emotion classes, minimizes subjective overfitting, and generalizes well to unseen samples. Quantitative and qualitative analyses were employed to compare the proposed work to a number of EEG generalization techniques such KPCA, VMD, and MIDA. In addition, an ablative experiment was performed to test the contributions of the individual components of the architecture. The findings of the experiments are summarized as follows:

- BiVDANN exhibits superior subject-independent classification performance among the context-generalization methods tested for both the SEED and DEAP datasets (see Table 3 and Table 4)
- The embeddings learned by BiVDANN exhibit both strong subject-invariance and a robust Gaussian distribution, allowing it to better accommodate unseen subjects (see Table 5 and Table 6)
- Ablation tests show that both VAE and DANN components contribute significantly to the robustness of the model, and that lateralization could be a significant contributor to identifying emotions in EEG (see Table 7 and Table 8)

Future works may benefit from the modular structure of the proposed method by extending any of its existing capabilities. For instance, it may be possible to improve the cross-dataset performance by employing additional context-based regularizers to minimize domain drift between datasets. Another direction could be to improve the processing of input features, e.g., by adding components for discovering complex spatial-temporal features equivalent to ICA or DE. Additionally, it may be of interest to include other signal modalities, such as ECG or GSR, together with EEG in order to find features unique to each but complementary to each other. Conversely, a weakness of the proposed work is its black-box nature, which makes it difficult to directly assess the features discovered. The large number of nonlinear components and varied types of parameter interactions makes it difficult to understand precisely how the trained model makes its decisions [64]. While DNN parameter visualization methods exist [64], they are generally geared more toward image-based models. Additionally, while there have been attempts to visualize deep EEG features [8], this required model architectures designed specifically for the visualization task. Future studies could find ways to combine these techniques to discover generalizable features that may be applied to manual EEG analysis. Finally, as more data becomes available or as more sophisticated noise rejection techniques are discovered, it may be possible to extend the method to a full end-to-end model by combining it with low-level EEG time-frequency feature extractors proposed by other ongoing works.

## Figures and Tables

**Table 1:** Summary of Related EEG Domain Adaptation and Generalization Methods.

Method	Algorithm Type	Domain Invariance	Adaptation Type	Distribution
TCA [24]	Kernel-based	MMD	Binary domain	Centered distribution
MIDA [25]	Kernel-based	HSIC	Multi-domain	Centered distribution
WGAN [28]	Deep Learning	Wasserstein distance	Binary domain	None
KPCA [32]	Kernel-based	Principal components	None	Centered distribution
VMD [33]	Signal Decomposition	IMF decomposition	None	Approx. Gaussian
VAE [35]	Deep Learning	None	None	Gaussian
SAAE [36]	Deep Learning	MMD	Binary domain	None
DANN [37]	Deep Learning	Adversarial training	Multi-domain	None
BiDANN [14]	Deep Learning	Adversarial training	Binary domain	None
BiVDANN [Ours]	Deep Learning	Adversarial training	Multi-domain	Gaussian

**Table 2:** BiVDANN Implementation Details.

Component	Input (Dims)	Components (Dims)	Output (Dims)
*Unilateral VAE*	*Topograph*: (32,64)	*Encoder*: 3 × [Conv2D(3 × 3) + BatchNorm] *VAE Embedding*: [Dense(512) + Mean: Dense(64) + SD: Dense(64) + z: Dense(64)] *Decoder*: 3 × [Conv2DTranspose(3 × 3) + BatchNorm]	*Unilateral Embedding*: (64)
*Bilateral Feature Extractor*	*Unilateral Embeddings*: 2 × (64)	[Concat + 2 × [Conv2D(2 × 2) + BatchNorm] + 2 × [Dense(128) + BatchNorm]]	*Bilateral Embedding*: (128)
*Emotion Classifier*	*Bilateral Embedding*: (128)	2 × [Dense(256) + BatchNorm] + 1 × [Dense(64) + BatchNorm] + Dense(Softmax)	*Emotion prediction*: (# of classes)
*Adversarial Subject Classifier*	*Bilateral Embedding*: (128)	GradientReversal + 2 × [Dense(256) + BatchNorm] + 1 × [Dense(64) + BatchNorm] + Dense(Softmax)	*Subject prediction*: (# of subjects)

**Table 3:** SEED Results (3-class).

	CNN	Resnet-50	KPCA+DNN	VMD+DNN	MIDA+DNN	BiVDANN
Mean	49.78%	52.31%	51.66%	58.45%	60.14%	63.28%
SD	5.09%	4.51%	6.67%	8.17%	4.51%	6.45%

**Table 4:** DEAP Results (2-class).

	CNN	Resnet-50	KPCA+DNN	VMD+DNN	MIDA+DNN	BiVDANN
Mean	51.64%	52.14%	55.31%	57.78%	60.97%	63.52%
SD	5.20%	7.32%	9.14%	6.75%	5.57%	5.21%

**Table 5:** SEED embedding visualizations for the tested models. Different colors correspond to their respective emotion class (negative, neutral, positive) or unique subject. The axis values represent the arbitrary 2D feature mapping performed by the dimension reduction algorithms.

Model	t-SNE		MDS	
	Classes	Subjects	Classes	Subjects
CNN	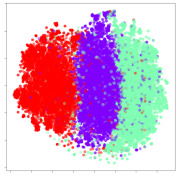	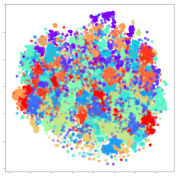	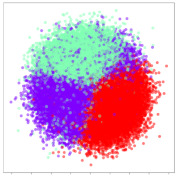	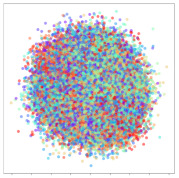
Resnet-50	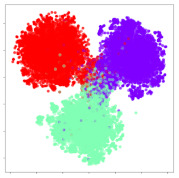	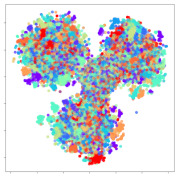	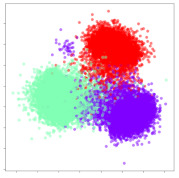	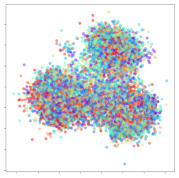
VMD	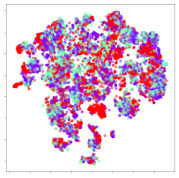	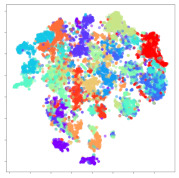	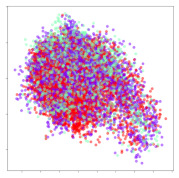	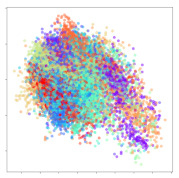
MIDA	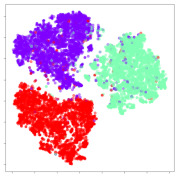	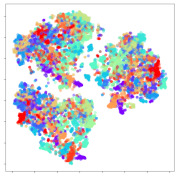	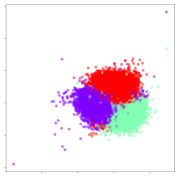	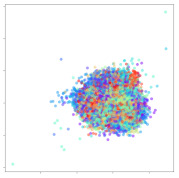
BiVDANN	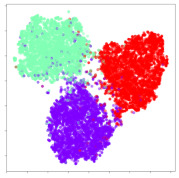	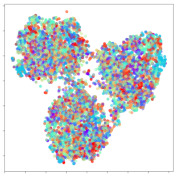	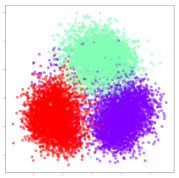	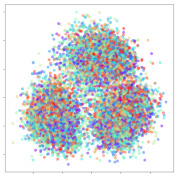
	**KPCA**			
	**Classes**		**Subjects**	
	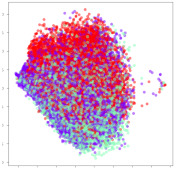		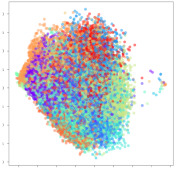	

**Table 6:** DEAP embedding visualizations for the tested models. Different colors correspond to their respective emotion class (negative, positive) or unique subject. The axis values represent the arbitrary 2D feature mapping performed by the dimension reduction algorithms.

Model	t-SNE		MDS	
	Classes	Subjects	Classes	Subjects
CNN	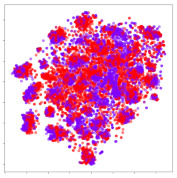	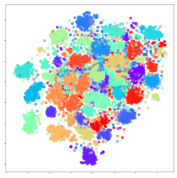	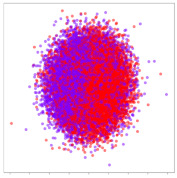	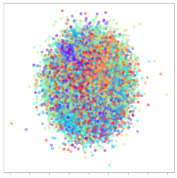
Resnet-50	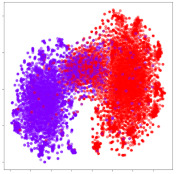	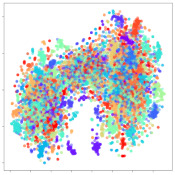	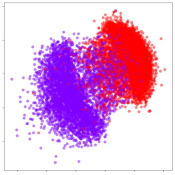	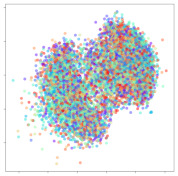
VMD	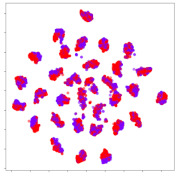	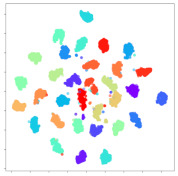	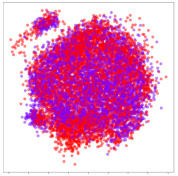	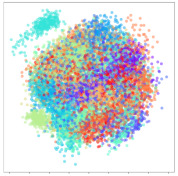
MIDA	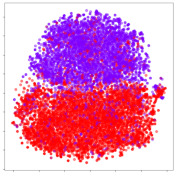	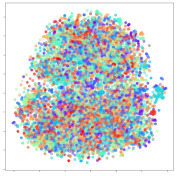	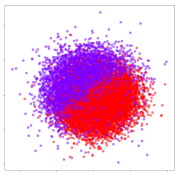	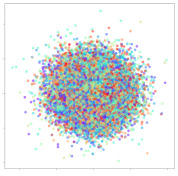
BiVDANN	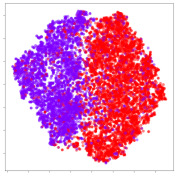	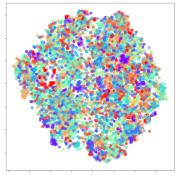	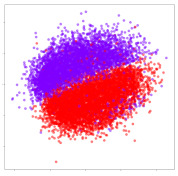	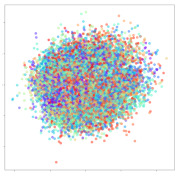
	**KPCA**			
	**Classes**		**Subjects**	
	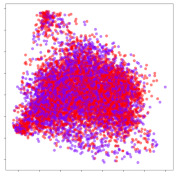		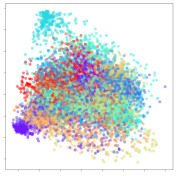	

**Table 7:** SEED Results (3-class).

	BiCNN	BiVAE	BiDANN	L-VDANN	R-VDANN	BiVDANN
Mean	52.02%	53.41%	60.71%	57.49%	58.54%	63.28%
SD	5.29%	3.80%	5.33%	6.11%	5.66%	6.45%

**Table 8:** DEAP Results (2-class).

	BiCNN	BiVAE	BiDANN	L-VDANN	R-VDANN	BiVDANN
Mean	52.15%	58.08%	61.59%	60.69%	62.10%	63.52%
SD	6.42%	6.32%	5.09%	4.11%	5.45%	5.21%

## Data Availability

The datasets analyzed in this study are available through their respective respository pages. The SEED datset is available upon request to its authors through the SJTU BCMI repository page: http://bcmi.sjtu.edu.cn/home/seed/index.html (accessed on 4 March 2021). The DEAP dataset is available upon request to its authors through the QMUL DEAP repository page: http://www.eecs.qmul.ac.uk/mmv/datasets/deap/ (accessed on 4 March 2021).
